# Identification of a Transcription Factor Controlling pH-Dependent Organic Acid Response in *Aspergillus niger*

**DOI:** 10.1371/journal.pone.0050596

**Published:** 2012-12-12

**Authors:** Lars Poulsen, Mikael Rørdam Andersen, Anna Eliasson Lantz, Jette Thykaer

**Affiliations:** Department of Systems Biology, Center for Microbial Biotechnology, Technical University of Denmark, Lyngby, Denmark; Universidade de Sao Paulo, Brazil

## Abstract

Acid formation in *Aspergillus niger* is known to be subjected to tight regulation, and the acid production profiles are fine-tuned to respond to the ambient pH. Based on transcriptome data, putative trans-acting pH responding transcription factors were listed and through knock out studies, mutants exhibiting an oxalate overproducing phenotype were identified. The yield of oxalate was increased up to 158% compared to the wild type and the corresponding transcription factor was therefore entitled Oxalic Acid repression Factor, OafA. Detailed physiological characterization of one of the *ΔoafA* mutants, compared to the wild type, showed that both strains produced substantial amounts of gluconic acid, but the mutant strain was more efficient in re-uptake of gluconic acid and converting it to oxalic acid, particularly at high pH (pH 5.0). Transcriptional profiles showed that 241 genes were differentially expressed due to the deletion of *oafA* and this supported the argument of OafA being a trans-acting transcription factor. Furthermore, expression of two phosphoketolases was down-regulated in the *ΔoafA* mutant, one of which has not previously been described in fungi. It was argued that the observed oxalate overproducing phenotype was a consequence of the efficient re-uptake of gluconic acid and thereby a higher flux through glycolysis. This results in a lower flux through the pentose phosphate pathway, demonstrated by the down-regulation of the phosphoketolases. Finally, the physiological data, in terms of the specific oxygen consumption, indicated a connection between the oxidative phosphorylation and oxalate production and this was further substantiated through transcription analysis.

## Introduction

*Aspergillus niger* is an industrially important organism, used as cell factory of a wide range of commercial enzymes as well as productions of million tons of citric acid [1]. Due to the significance of *A. niger* in the biotech industry, strain improvement is a key component in process optimization. Traditionally, it has been approached by genetic engineering of a single or few metabolic genes; however, this strategy struggles to overcome the superjacent regulation thus the outcome has frequently shown to be of limited success. Another strategy entails of direct manipulation of transcription factors (TFs), since these proteins have the potential of controlling several fluxes in an organism. Modulation of TFs as a strategy for metabolic engineering has been demonstrated by Schuurmans *et al.* 2008, where the authors deleted one TF and overexpressed another to improve ethanol production in *Saccharomyces cerevisiae* [2]. A different approach is to use site directed mutagenesis on a TF, an approach that was applied to improve ethanol tolerance and production in *S. cerevisiae* [3]. Both methods have been applied in prokaryotes as well as in unicellular eukaryotes, but to our knowledge have not been tested in multicellular eukaryotes. One explanation may be that the genome sequence for *S. cerevisiae* have been public available since 1996 [4], whereas the first filamentous fungal genome was released less than ten years ago, with the genome of *Neurospora crassa* being the earliest to be publically available [5]. Another challenge is the complexity of the regulatory networks caused by the large genomes in multicellular eukaryotes, illustrated by the number of increasing TFs with increasing genome size e.g. *E. coli* has 48 [6] whereas *A. niger* has approximately 1000 [7] and only a few of them have been functionally characterized.

Transcription factors can be grouped as cis- or trans-acting. Cis-acting TFs are characterized by their local response, inducing an entire cluster including the transcription factor itself as in the case of many secondary metabolite clusters [8], [9], [10]. The other type of transcription factors, trans-acting, can regulate genes from a different region/chromosome of the genome than the region it was transcribed from itself as in the case of protease production, e.g. PrtT [11]. This transcription factor, located on chromosome VI, controls proteases scattered throughout the genome, regulating the majority of the extracellular protease response in *A. niger.* Deleting *prtT* significantly lowers the protease activity without a noteworthy effect on the physiology of the fungus [11]. Considering the nature of organic acid production by *A. niger* being highly dependent on pH of the culture medium [12], [13], [14], we hypothesize, that acid production is mediated through a trans-acting transcription factor response.

Based on transcriptome data from a previous study where *A. niger* was cultivated at three different pH set points [15], we identified a list of putative trans-acting pH responding transcription factors which formed the basis for sequential knockout studies. In the following screening process, particularly one set of mutants exhibited an elevated acidification of the media, which corresponded to increased oxalate production. The responsible transcription factor was therefore entitled OafA (oxalic acid repression factor). One of the deletion mutants, *ΔoafA,* and the reference strain, were subjected to detailed physiological characterization.

## Materials and Methods

### Fungal strains

*A. niger* ATCC 1015 was used as wild type strain (obtained from the IBT collection as IBT 28639). The *ΔoafA* strains were generated from the ATCC 1015 strain. All strains were maintained as frozen spore suspensions at −80°C in 20% glycerol.

### Media

Transformation medium: 182.2 g/L sorbitol, 10 g/L glucose monohydrate, 6 g/L NaNO_3_, 0.52 g/L KCl, 0.52 g/L MgSO_4_ · 7H_2_O, 1 mL/L of 1% thiamine solution, 1 mL/L trace element solution. Trace element solution: 22 g/L ZnSO_4_ · 7 H_2_O, 11 g/L H_3_BO_3_, 5 g/L MnCl_2_ · 4 H_2_O, 5 g/L FeSO_4_ · 7 H_2_O, 1.7 g/L CoCl_2_ · 6 H_2_O, 1.6 g/L CuSO_4_ · 5 H_2_O, 1.5 g/L Na_2_MoO_4_ · 2 H_2_O, 50 g/L Na_4_EDTA.

Czapek yeast extract (CYA) media: 30 g/L Sucrose, 5 g/L Yeast extract, 3 g/L NaNO_3_, 1 g/L K_2_HPO_4_, 0.5 g/L MgSO_4_ · 7 H_2_O, 0.5 g/L KCl, 0.01 g/L FeSO_4_ · 7H_2_O, 15 g/L Agar, 1 mL/L trace element solution. 0.4 g/L CuSO_4_ · 5 H_2_O, 0.04 g/L Na_2_B_2_O_7_ · 10 H_2_O, 0.8 g/L FeSO_4_ · 7 H_2_O, 0.8 g/L MnSO_4_ · H_2_O, 0.8 g/L Na_2_MoO_4_ · 2 H_2_O, 8.0 g/L ZnSO_4_ · 7 H_2_O. pH adjusted to 6.2 prior to autoclavation.

Minimal screenings medium: 10 g/L glucose monohydrate, 6 g/L NaNO_3_, 0.52 g/L KCl, 0.52 g/L MgSO_4_ · 7H_2_O, 1 mL/L of 1% thiamine solution, 1 mL/L trace element solution. Trace element solution: 22 g/L ZnSO_4_ · 7 H_2_O, 11 g/L H_3_BO_3_, 5 g/L MnCl_2_ · 4 H_2_O, 5 g/L FeSO_4_ · 7 H_2_O, 1.7 g/L CoCl_2_ · 6 H_2_O, 1.6 g/L CuSO_4_ · 5 H_2_O, 1.5 g/L Na_2_MoO_4_ · 2 H_2_O, 50 g/L Na_4_EDTA. As buffer 19.52 g/L 2-(N-morpholino)ethanesulfonic acid (MES) was used. pH was adjusted to 6.0 prior to autoclavation.

Complex screening medium (Watman medium): 30 g/L Sucrose, 5 g/L Corn steep liquor, 2 g/L Yeast extract, 3 g/L Peptone, 2 g/L Glucose, 2 g/L NaNO_3_, 1 g/L K_2_HPO_4_ · 3 H2O, 0.5 g/L MgSO_4_ · 7 H2O, 0.05 g/L FeSO_4_ · 7 H2O, 0.2 g/L KCl, 1 mL/L Trace metal solution. Trace element solution: 22 g/L ZnSO_4_ · 7 H_2_O, 11 g/L H_3_BO_3_, 5 g/L MnCl_2_ · 4 H_2_O, 5 g/L FeSO_4_ · 7 H_2_O, 1.7 g/L CoCl_2_ · 6 H_2_O, 1.6 g/L CuSO_4_ · 5 H_2_O, 1.5 g/L Na_2_MoO_4_ · 2 H_2_O, 50 g/L Na_4_EDTA. As buffer 19.52 g/L 2-(N-morpholino)ethanesulfonic acid (MES) was used. pH was adjusted to 6.0 prior to autoclavation.

Batch cultivation medium: 20 g/L glucose, 7.3 g/L (NH_4_)_2_SO_4_, 1.5 g/L KH_2_PO_4_, 1.0 g/L MgSO_4_ · 7 H_2_O, 1.0 g/L NaCl, 0.1 g/L CaCl_2_, 0.1 mL Antifoam 204 (sigma), 1 mL/L trace element solution. Trace element solution: 0.4 g/L CuSO_4_ · 5 H_2_O, 0.04 g/L Na_2_B_2_O_7_ · 10 H_2_O, 0.8 g/L FeSO_4_ · 7 H_2_O, 0.8 g/L MnSO_4_ · H_2_O, 0.8 g/L Na_2_MoO_4_ · 2 H_2_O, 8.0 g/L ZnSO_4_ · 7 H_2_O.

### Preparation of inoculum

Conidia were propagated on CYA media plates and incubated for 5 to 7 days at 30°C before being harvested with 2 times 10 ml 0.9% NaCl and filtered through mira cloth and washed twice with 0.9% NaCl. Fermentations were initiated by conidia inoculation to a final concentration of 2·10^9^ spores/L

### Target selection

A previous study [15] identified 6228 genes which react significantly (p<0.05) to pH over three different conditions (pH. 2.5, 4.5, and 6.0). From this set of data, we examined all genes that react in a progressive manner over the values, either increasing or decreasing with pH, and extracted all predicted transcription factors in this set.

### PCR amplification

All PCR reactions were carried out using the high fidelity Phusion polymerase from Finnzymes at standard conditions with HF-buffer.

### Gene deletion

All DNA insertions into the *A. niger* genome were performed using protoplasts and PEG transformation. The deletion strains were constructed using PCR-generated bipartite gene targeting substrates [16]. Each part of the bipartite substrate consisted of a targeting fragment and a marker fragment, all of which were amplified individually by PCR using the primer pairs presented in Table 1. Hygromycin phosphotransferase gene (hph) marker cassette was amplified from plasmid pCB10003 [17] as template DNA.

**Table 1:** Primers used for deletion of *oafA* in *A. niger*.

Primer	Sequence
Upst_OAFA_F	TATGACGTGCGGGTATTCGAG
Upst_OAFA_R	gatccccgggaattgccatgTAACTTCAGTAGATCGCCCAGC
Dwst_OAFA_F	ggactgagtagcctgacatcTTAGCAGGGGCGAATAATGC
Dwst_OAFA_R	CGAATGAACAACAGCAGGATG
Upst_Hyg_F	catggcaattcccggggatcGCTGGAGCTAGTGGAGGTCA
Upst-HygR-N	CTGCTGCTCCATACAAGCCAACC
Dwst-HygF-N	GACATTGGGGAGTTCAGCGAGAG
Dwst-Hyg_R	gatgtcaggctactcagtccCGGTCGGCATCTACTCTATT

Lower-case letters indicate overlapping genetic elements used for fusion PCR.

### Oligonucleotide PCR primers

The oligonucleotides used for the strain construction of Δ*oafA*, can be found in Table 1.

### Southern blotting

**Figure 1:**
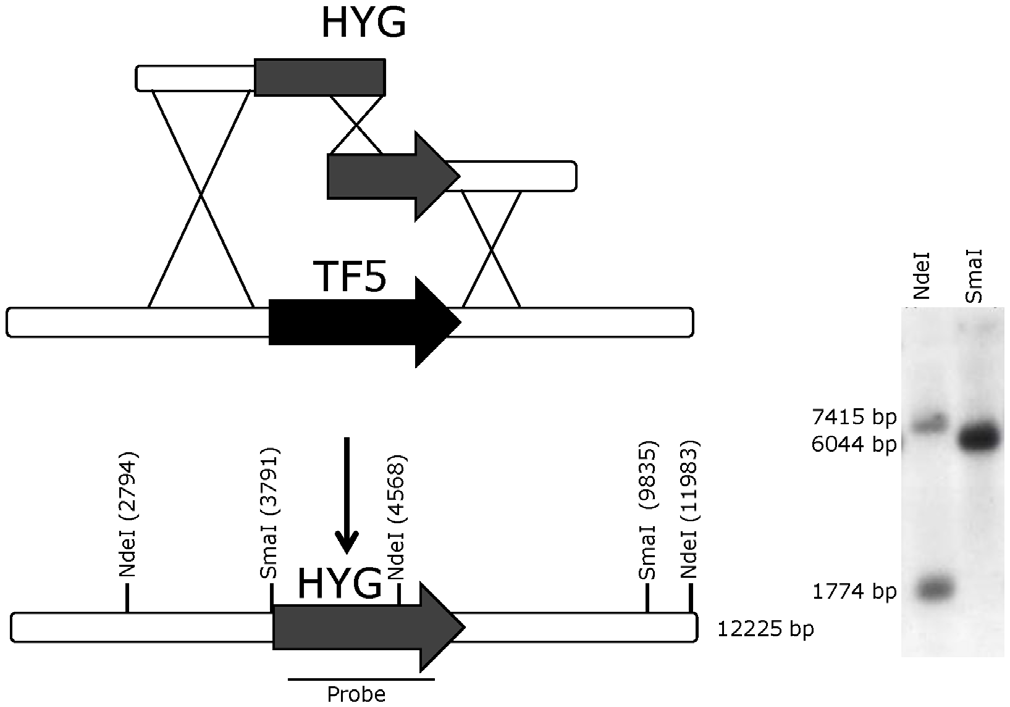
Graphical illustration of the gene deletion procedure exemplified with by insertion of hygromycin resistance marker into the *oafA* locus. (A) Bipartite substrate, locus and predicted resultant genomic locus. (B) Southern analysis of transformants for site specific integration of the construct. Genomic DNA was digested with either NdeI or SmaI. The position of the probe used is shown in (A).

1.5 µg genomic DNA was isolated and digested with appropriate restriction enzymes (SmaI and NdeI). Sequence information for restriction digest of the target loci was obtained from the *A. niger* ATCC 1015 genome sequence [18] from the US Department of Energy Joint Genome Institute (http://genome.jgipsf.org/Aspni1/). Blotting was done according to standard methods [19], using RapidHyb hybridization buffer (Amersham Pharmacia) for probing. The target locus was detected by probing with the labeledmarker gene PCR fragment. The probes were radioactively labeled with α-32P-dCTP by random priming using Rediprime II kit (GE Healthcare). For a graphical representation of the gene deletion strategy, see Figure 1.

### Cultivations

#### Static cultivations

Fresh conidia were added to 8 mL of minimal- or complex screenings medium in a 50 mL sterile falcon tube (BD Biosciences) to a final concentration of 1·10^3^ conidia/mL and incubated without agitation at 30°C for 5 days. At the end of the experiment, extracellular samples for pH and HPLC-measurements were collected.

#### Batch cultivations

Batch cultivations were performed in 2 L Sartorius fermenters with a working volume of 1.5 L, equipped with two Rushton six-blade disc turbines. The bioreactor was sparged with air, and the concentrations of oxygen and carbon dioxide in the exhaust gas were measured in a gas analyzer (1311 Fast response Triple gas, Innova combined with multiplexer controller for Gas Analysis MUX100, B. Braun Biotech International (Melsungen, Germany)). The temperature was maintained at 30°C during the cultivation and pH was controlled by automatic addition of 2 M NaOH and 1 M HCl, respectively. Initial conditions in the bioreactor were pH: 3.0; stirring rate: 100 rpm; and aeration: 0.2 volumes of air per volume of fluid per minute (vvm). After germination, the stirring rate was gradually increased to 800 rpm and the air flow to 1 vvm. The pH was adjusted to 2.5 or 6.0 with addition of 2 M NaOH or 1 M HCl over 2 hours.

#### Chemostat cultivations

The chemostat cultivations were initiated as batch cultivations and in late exponential phase, supply of additional substrate through the feed was initiated. The feed medium was similar to the batch medium with the exception of the glucose concentration being 8 g/L. Pumps were controlled to a rate of 150 g/h (D = 0.1 h^−1^). The first steady state, pH 2.5, was obtained after five residence times (50 hours), whereas the second steady state was achieved after three additional residence times (30 hours).

The pH shift to the second steady state was performed over five hours to avoid a morphological shift towards pellet formation.

### Cell dry weight determination

The cell mass concentration on a dry weight basis was determined by the use of nitrocellulose filters with a pore size of 0.45 µm (Osmonics, Minnetonka, MN, USA). Initially, the filters were pre-dried in a microwave oven at 150 W for 20 min, and then weighed. A known weight of cell culture was filtered, and the residue was washed with distilled water. Finally, the filter was dried in the microwave at 150 W for 20 min and the dry weight was determined.

### Quantification of extracellular metabolites

#### HPLC analysis

For quantification of the extracellular metabolites, a culture sample was taken and immediately filtered through a 0.45 µm-pore-size nitrocellulose filter (Osmonics). The filtrate was frozen and kept at −20°C until analysis. Glucose, oxalate, citrate, gluconate, glycerol and acetate concentrations were detected and quantified by refractive index and UV using an Aminex HPX-87H cationic-exchange column (BioRad, Hercules, CA, USA) eluted at 35°C, with 5 mM H2SO4 at a flow rate of 0.6 mL min^−1^.

#### Glucose assay

The applied HPLC method is unable to separate glucose and gluconate. Therefore, in samples where both glucose and gluconate were present, gluconate was quantified based on the UV-spectrum from the HPLC-analysis, whereas glucose was determined using a glucose enzymatic assay (Horiba ABX, Montpellier, France). This assay was based on NAD^+^ through a coupled reaction with glucose-6-phosphate dehydrogenase and formation of NADH was determined spectrophotometrically by measuring the increase in absorbance at 340 nm.

### Transcription analysis

#### Sampling

For gene expression analysis, mycelium was harvested at steady state by filtration through sterile Mira-Cloth (Calbiochem, San Diego, CA, USA). Liquid was quickly removed from the mycelium by squeezing, and it was then subsequently frozen in liquid nitrogen. Samples were stored at −80°C until RNA extraction.

#### Extraction of total RNA

Total RNA was extracted from 40 to 50 mg of frozen mycelium as described in [15] and the quantity was determined using a Qubit 2.0 Fluorometer (Invitrogen). The total RNA was stored at −80°C until further processing.

#### cRNA and microarray

150 ng of total RNA in 1.5 µl was labeled according to the One Color Labeling for Expression Analysis, Quick Amp Low Input (QALI) version 6.5 May 2010 from Agilent Technologies. Yield and specific activity was determined on a NanoDrop ND-1000 and verified on Qubit 2.0. 1.65 µg labeled cRNA was fragmented at 60°C on a heating block and the cRNA was prepared for hybridization according to the QALI protocol.

100 µl sample was loaded on a 4×44 Agilent Gasket Slide situated in an Agilent Technologies hybridization chamber. The 4×44 Array was placed on top of the Gasket Slide. The Array was hybridized at 65°C for 17 hours in an Agilent Technologies Hybridization oven. The array was washed following the QALI protocol and scanned in a G2505C Agilent Technologies Micro Array Scanner.

#### Analysis of transcriptome data

The raw array signal was processed by first removing the background noise using the normexp method, and signal between arrays made comparable using the quantile normalization method, as implemented in the Limma package [20]. Multiple probe signals per gene were summarized into a gene-level expression index using Tukey's medianpolish [21].

Statistical analysis was applied to determine genes subjected to differential transcriptional regulation. The limma package [20] was used to perform moderated *t* tests between sets of biological replicates from each pH level. Empiric Bayesian statistics were used to moderate the standard errors within each gene, and Benjamini-Hochberg's method [22], to adjust for multitesting. A cut-off value of adjusted *P*<0.05 was set to assess statistical significance.

### Gene ontology enrichment analysis

Significantly regulated subsets of genes were examined for GO-term enrichment by using Cytoscape with BiNGO plugin (Maere, Heymans et al. 2005). GO-term assignments were based on automatic annotation of the *A. niger* ATCC 1015 v3.0 gene models. The significance level was selected to P<0.05 and calculated using hypergeometric testing with Benjamini & Hochberg correction.

## Results

*A. niger* is known for its acid formation and recent studies have shown that a up to 6228 genes are influenced by ambient pH and the physiological response results in an acid production profile that ensures optimal acidification of the surrounding medium [15]. Based on these results, it was hypothesized that the acid formation response is mediated by trans-acting TFs, since the affected genes were scattered across the entire genome of *A. niger*. Further analysis of the data from [15] resulted in a set of predicted transcription factors. The data was sorted into 17 clusters. In the largest cluster, which consisted of 2814 genes, a large number of housekeeping genes were identified. The main function of the genes in this cluster was considered to be growth or stress related and therefore not directly regulated by pH. Based on this, the cluster was excluded. In the resulting subset, now containing 3414 genes, the putative cis-acting transcription factors were predicted and removed. The predictions were performed using an in-house method based on their co-regulation with closely positioned biosynthetic clusters (data not shown). Finally, the trans acting transcription factors were ranked based on p-values.

The genes encoding the predicted TFs were individually deleted through a bipartite gene knockout approach [16] using hygromycin as dominant marker, Figure 1.A. The gene deletion was verified using PCR and resulting TF mutants were initially screened for an altered acid formation profile in static cultures (liquid cultures incubated without shaking). Three transformants per knockout were analyzed to ensure the phenotype was originated from a monogenetic effect. The assay simply relied on measurement of pH in the ambient medium. Particularly, one set of transformants showed an interesting phenotype as a significant decrease in pH was measured compared to the pH value in the wild type culture (data not shown). Subsequent HPLC analysis showed an increased oxalate formation, Table 2. To validate for ectopic insertions, Southern analysis was carried out. For this analysis, the probe was designed so digestion with NdeI resulted in 2 bands of 1774 bp and 7415 bp respectively, whereas SmaI digestion resulted in only one band of 6044 bp. From the Southern blot, Figure 1.B, only correct sized bands were observed, thus the HygR cassette had been integrated only at the right position in the genome of *A. niger* ATCC 1015. The corresponding transcription factor was entitled OafA (oxalic acid repression factor).

**Table 2:** Oxalate concentration in static cultures pH 6.0.

	WT		*ΔoafA*	
		Transformatant A	Transformant B	Transformant C
	Oxalate g/L	Oxalate g/L	Oxalate g/L	Oxalate g/L
**Minimal medium**	1.2	2. 8	2.7	2.3

### Physiological characterization of the *ΔoafA-*strain

The constructed *ΔoafA* strain was subjected to detailed physiological characterization to explore the cellular performance of the oxalate overproducing strain from a quantitative perspective.

#### Batch cultures

**Figure 2:**
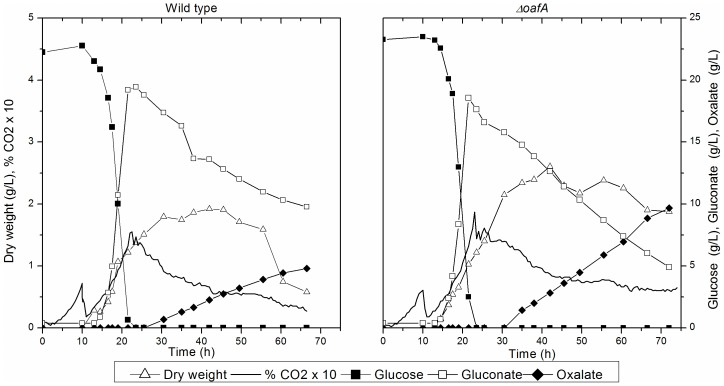
Representative profiles of the biomass concentration, sugar concentration, carbon dioxide formation and acid formation during batch cultivations at pH 6.0 with the WT-strain (left) and and the *ΔoafA* strain. The maximum specific growth rate was estimated trough a logarithmic plot of the biomass concentration as a function of time. Yield coefficients were calculated as overall yields based on the accumulated biomass or metabolite concentration in stationary phase related to the amount of consumed glucose. The volumetric oxalate formation rate was estimated as the slope of a linear regression of the oxalate titer as a function of time.

The physiology of the wild type strain and the *ΔoafA* strain was compared in pH-controlled batch cultivations, in duplicates. *A. niger* has been reported to produce a range of different acids in high amounts at pH 6.0 [12], [15]. Thus, by performing the batch cultures at this pH the acid profile together with the level of acid formation were challenged in the constructed *ΔoafA* strain. Representative profiles of the biomass concentration, sugar concentration, carbon dioxide formation and acid formation during these batch cultivations are shown in Figure 2.

Characteristic for both the wild type and the *ΔoafA* strain was that the majority of glucose in the exponential growth phase was converted into gluconate and biomass, where gluconate formation accounted for approximately 90% of the carbon conversion. After complete utilization of glucose, gluconate was metabolized resulting in mainly oxalate formation, but citrate was also detected in both set of cultures, Figure 2. The maximum specific growth rates of the two strains were 0.23±0.02 h^−1^ and 0.25±0.02 h^−1^ for the WT and the *ΔoafA* strain respectively, so deleting *oafA* showed no effect on the growth rate. However, a notable difference was observed in the formation of oxalate as the yield of oxalate on glucose was increased by 87% from 0.13 Cmol Cmol^−1^ to 0.25 Cmol Cmol^−1^ and the volumetric oxalate formation rate was doubled from 0.11±0.02 gOx (L h)^−1^ in the WT to 0.22±0.00 gOx (L h)^−1^ in the *ΔoafA* strain.

An interesting observation from the results of the batch cultivations was that acid production, except for gluconate formation, was mainly measured in stationary phase after glucose completion. From Figure 2 it appears that during stationary phase the cells are converting gluconate to oxalate and oxalate formation is therefore decoupled from growth.

#### Characterization of ΔoafA in chemostat cultivations

**Figure 3:**
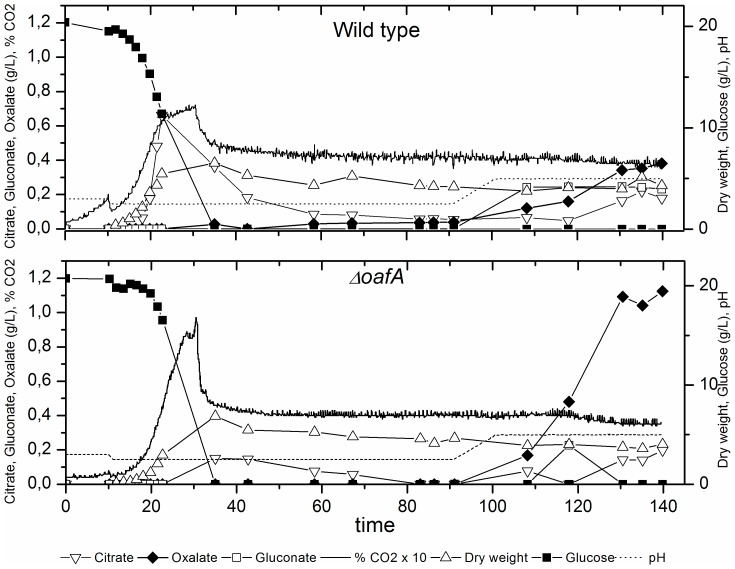
Representative profiles of chemostat cultivations for wild type (top) and *ΔoafA* (bottom). Two steady states were obtained for each chemostat cultivation performed.

To compare the acid production profiles of *ΔoafA* and the wild type during growth at both high and low pH, carbon limited chemostat cultivations were carried out. An important feature in the presented characterization was the application of chemostat cultures, as it can be difficult to obtain and maintain steady states working with filamentous fungi, because of the complex morphology of the cells [23]. However, in this study steady states were obtained for both strains as well as for the two selected pH levels, Figure 3. The experimental setup was designed in a way that two steady states were obtained for each chemostat performed. An advantage of such a design is the possibility of attaining data from two distinct pH values using a single chemostat setup. Moreover, morphological issues such as pellet formation have been observed in transition from batch to chemostat at high pH (data not shown). To avoid pellet formation, the batch phase and the first steady state were at pH 2.5, whereas pH 5.0 was chosen for the second steady state, illustrated in Figure 3.

Figure 3 shows that for both strains, the main products in the exponential phase were biomass and CO_2_ at comparable yields. However, low citrate production was observed in the wild type, a product not detected in *ΔoafA*. During the first steady state at pH 2.5, achieved at 73 h (5 retention times), the mutant had an unchanged product profile with identical quantities in comparison with the wild type. The second steady at state occurred at 126 hours, 3 retention times after pH was changed to pH 5.0. As it was evident in the high pH batch cultivation, *ΔoafA* had a highly increased oxalate formation (158%) compared to WT, whereas both citrate and gluconate formation were reduced. A summary of growth and yield coefficients can be found in Table 3.

**Table 3:** Physiological coefficients from chemostat cultivations.

		Wild-type (Cmol/Cmol)	*ΔoafA* (Cmol/Cmol)	Difference
Batch phase, pH 2.5	Y_sx_	0.820±0.026	0.803±0.026	−2%
	Y_s,gluconate_	0	0	0%
	Y_s,oxalate_	0	0	0%
	Y_s,citrate_	0.063±0.027	0	−100%
	Y_s,CO2_	0.225±0.005	0.232±0.007	3%
	Y_so_	0.283±0.05	0.282±0.0 10	0%
Chemostat, pH 2.5	Y_sx_	0.666±0.019	0.685±0.014	3%
	Y_s,gluconate_	0	0	0%
	Y_s,oxalate_	0	0	0%
	Y_s,citrate_	0	0	0%
	Y_s,CO2_	0.330±0.002	0.313±0.019	−5%
	Y_so_	0.043±0.004	0.036±0.005	−16%
Chemostat, pH 5.0	Y_sx_	0.679±0.024	0.593±0.005	−13%
	Y_s,gluconate_	0.034±0.003	0	−100%
	Y_s,oxalate_	0.034±0.004	0.088±0.003	158%
	Y_s,citrate_	0.017±0.005	0.006±0.011	−62%
	Y_s,CO2_	0.335±0.021	0.273±0.032	−19%
	Y_so_	0.046±0.004	0.030±0.004	−35%

From Table 3 it is apparent that the main different between the *ΔoafA* mutant and the wild type strain is increased oxalate formation in the *ΔoafA* mutant at the expense of gluconate, particularly in the second steady state at pH 5.0. The validity of the observed oxalate overproducing phenotype in the *ΔoafA* mutant is substantiated by the carbon balances, which closes for each distinctive phase.

The *ΔoafA* thus exhibits an oxalate overproducer phenotype at high pH in both the reported batch and chemostat culture, as well as in the initial screening. To investigate the transcriptional aspects of this phenotype and to obtain further insight to OafA's function as a transcription factor, biomass samples were taken from all biological triplicates at steady state conditions (12 samples in total) and subjected to transcriptome analysis.

### Transcriptome analysis

Data from the biological triplicates of the two strains at pH 2.5 and 5.0 were statistically analyzed, and genes that were significantly regulated (Benjamini-Hochberg corrected Bayesian P values<0.05) in pair-wise comparisons between two strains were identified across pH. The raw data of the expression levels of the entire *A. niger* genes analyzed are presented in the Table S1 together with complete lists of the genes differentially expressed in the two strains at different pH.

Comparing the wild type with the *oafA* mutant at the first steady state (pH 2.5), a notably low number of significantly regulated genes was identified. Seven genes with a significant change in expression level, where four have been annotated, were identified. They included: down-regulation of a putative lysophospholipase, a polyketide syntease and intriguingly oxaloacetate acetyl hydrolase (*oahA*), the enzyme converting oxaloacetate to oxalate.

At pH 5.0, 241 genes were significant changed in expression levels, 121 genes being up-regulated and 120 being down-regulated. A Gene Ontology overrepresentation analysis on the subset of up-regulated genes revealed an enrichment of 19 genes (p-value 3.43·10^−4^, cluster freq 19/64, genome freq. 818/6268) associated with transport processes and within this subset three of these putative transporters were annotated as hexose transporters.

Among the up-regulated genes, five TFs were found, where four were located next to putative secondary metabolite clusters. However, each TF showed a limited degree of co regulation with their nearby secondary metabolite cluster, predicted by an in-house method (data not shown). Additionally, two genes encoding for epigenic regulation, a RNA-dependent RNA polymerase (Gene ID: 176277) and a histone acetyltransferase (Gene ID: 53882) were also found to be up-regulated.

The phenotypic trait of the *ΔoafA* strain was shown to be a significant increase in oxalate formation. It was therefore surprising that the gene encoding *oahA* (oxalate dehydrogenase) was not up-regulated. Yet a slight up-regulation of a cytosolic malate dehydrogenase (MDH), the enzyme responsible for converting malate to oxaloacetate, was measured.

An ontology overrepresentation analysis on the subset of down-regulated genes showed an enrichment of 28 genes associated with oxidoreductase activity (p-value 1.58·10^−5^, cluster freq 28/52, total freq 1300/6268). Two of these genes, a NADH-dehydrogenase and monooxygenase were associated with the oxidative phosphorylation. A catalase and a chloroperoxidase were additionally found down-regulated, a further indication of reduced oxidative phosphorylation.

In addition, down-regulation of two phosphoketolases was detected. Gene ID 54814 contained zero introns, suggesting this gene could be of bacterial origin. Gene ID 197387 contained six introns indicating that it has evolved within the eukaryote kingdom. An alignment of the two protein sequences showed 88% query coverage and a max identity of 44% suggesting that they are not homologs. Down-regulation of acetate kinase (206885) and an Acyl-CoA syntase (129581), both enzymes responsible for converting acetate-phosphate into acetyl-CoA, was observed as well.

## Discussion

Deletion of the putative trans-acting transcription factor entitled oxalic acid repression factor, OafA, resulted in an oxalate overproducing strain. The physiology of the constructed strain was characterized in detail, in both batch and chemostat cultivations, and compared to wild type physiology. In addition, transcriptome analysis at steady state conditions at both high and low pH highlighted the transcriptional response caused by the deletion of *oafA*.

### Differences in acid production

The physiological characterization of the *ΔoafA* mutant showed an altered organic acid production profile at high pH compared to the wild type strain. All of the acid yields were affected, but in particular the oxalate yield increased (Figure 2, Figure 3 and Table 3). In the batch cultures, glucose was converted to gluconate with a similar specific yield of approximately 90% in the exponential phase, but the *ΔoafA* strain showed an improved reuptake and conversion of gluconate to oxalate. In the chemostat cultivation at pH 5.0, no gluconate was detected for the mutant and the oxalate yield on glucose was increased. Based on these results it may be argued that gluconate was produced with a similar rate in the mutant and the wild type, but the increased reuptake of gluconate and conversion to oxalate, argued from the batch results, leads to a complete re-consumption of gluconate and therefore no detectable level of this acid. The citrate formation was in both cultivation types found to be reduced by approximately 60%, however this was the least reproducible coefficient, hence it should be cautiously interpreted. An intriguing observation from the chemostat cultivations at pH 5.0 was the total acid yield, summarizing the yields of gluconate, citrate and oxalate on glucose, being similar for the two strains corresponding to 0.099 cmol cmol^−1^ for the wild type strain and 0.094 cmol cmol^−1^ for the *ΔoafA* mutant. This indicates that the capacity for acid production is the same for the two strains under the defined conditions, but deletion of the oxalic acid repression factor ensures a more efficient conversion of gluconate to oxalate. The acid response of *A. niger* has been argued to be an evolutionary selection for efficient acidification of the environment and gluconate does not contribute to a noteworthy acidification compared to oxalate and citrate [15]. The conversion of glucose to gluconate is therefore considered an efficient way of rapidly make glucose unavailable for competing organisms [12], [15] Thus, the constructed *ΔoafA* appears to be better at acidifying the ambient medium through efficient metabolization of gluconate.

### Transcriptome analysis

The physiological characterization provided insight to the profile and quantitative aspects of acid production in the constructed *ΔoafA* mutant compared to the wild type strain. However, details on how the deletion of *ΔoafA* affected the central carbon metabolism were limited, which is of high relevance since the main metabolic response appears to be more efficient utilization of gluconate in the constructed strain. Therefore, transcriptional profiling across both strains and pH were performed. Transcriptome analysis was carried out on biological material from the chemostat cultures. Using this type of setup, pH and the strains were the only variables and running the experiments in triplicates ensured high reproducibility, Table 3. Furthermore, at steady state conditions in a carbon limited culture it is possible to mimic the conditions at glucose depletion and simultaneously obtain both acid formation and growth. This enables discrimination of even low-fold differences in expression and has been successfully applied earlier in *Aspergillus oryzae* [24].

The number of differentially expressed genes identified in the transcription analysis at pH 5.0 was 241. Considering that this response is caused by deletion of a single gene, *oafA*, it appears remarkable that one putative trans-acting TF is regulating this amount of genes. An explanation of the high number of affected genes could be the up-regulation of two genes connected to epigenetic regulation, especially a putative histone acetyltransferase. Epigenetic regulation has attracted attention in connection with secondary metabolite production [25], [26], as it has been seen that certain secondary metabolism clusters are silent, but can be activated by deletion of the histone deacetylase HdaA, or inhibition of other fungal HDACs by trichostatin A [27].

Oxaloacetate hydrolase encoded by *oahA* is an important enzyme in oxalate production, as it catalyzes the conversion of oxaloacetate to oxalate and acetate. It was therefore a surprising discovery that no change in expression level of *oahA* was measured at high pH in the *ΔoafA* mutant, since this strain was found to be an oxalate overproducer. This indicates that *oahA* is either regulated after translation or *oahA* is not a rate-limiting step for oxalate formation. The latter appears to be more likely since the *K_m_* value for OahA is 0.004 mM [28] and *K_m_* for malate dehydrogenase is 0.09 mM [29] and an up-regulation of a cytosolic malate dehydrogenase was observed at high pH. This enzyme has previously been shown to play a key role in acid production [30], as over expression of malate dehydrogenase lead to increased citrate production as well as oxalate production. At low pH a significant down-regulation of *oahA* was measured, which was unexpected as the *oafA* mutant had an oxalic acid overproducing phenotype. However, as shown in Table 3 and supported by Ruijter *et al.* 1999 [12], oxalate was not detected at low pH and that makes the observation less important with respect to the *oafA* phenotype at low pH.

Another interesting observation was the up-regulation of transporters in the *ΔoafA* mutant at high pH, particularly sugar permeases. This could explain the lack of gluconate in these chemostats, since the increased reuptake of gluconate could be mediated by the higher permease activity. Despite this higher permease activity, no genes coding for glycolytic enzymes were to be found differentially regulated. This is however a consequence of the chemostats being glucose limited and the glucose uptake cannot be affected by the higher permease activity.

### The role of phosphoketolases in oxalate production

Deletion of *oafA* resulted in a metabolic shift in the central metabolism demonstrated by the down-regulation of both phosphoketolases (Gene ID 54814 and 197387) in the *ΔoafA* mutant. Two types of phosphoketolases have been described in the literature. Type 1 (EC 4.1.2.9) catalyzes, in the presence of inorganic phosphate, an irreversible cleavage of D-xylulose-5-phosphate into acetyl phosphate and glyceraldehyde-3-phosphate. Type 2 (EC 4.1.2.22) also termed fructose-6-phosphate phosphoketolase catalyzes as well in presence of phosphate, an irreversible cleavage of D-fructose 6-phosphate into acetyl phosphate and D-erythrose 4-phosphate. The activity of type 1 phosphoketolases is well characterized in a wide range of bacterial species and recently indications of this activity were found in *Penicillium chrysogenum* [31]. Type 2 activity has only been described within a relative low number of bacteria with bifidobacterium being most predominant [32].

An interesting finding in relation to Gene ID 197387 was that the open reading frame (ORF) of this gene was located next to the ORF of an acetate kinase. The close linkage of the ORFs of phosphoketolase and acetate kinase is found in many bacteria [33], and this argues for Gene ID 197387 being a D-xylulose 5-phosphate phosphoketolase. Which type Gene ID 54814 is encoding, can only be speculative since the only annotated gene in the vicinity is a hypothetical dehydrogenase with an interpro id suggesting a glucose/ribitol dehydrogenase activity.

**Figure 4:**
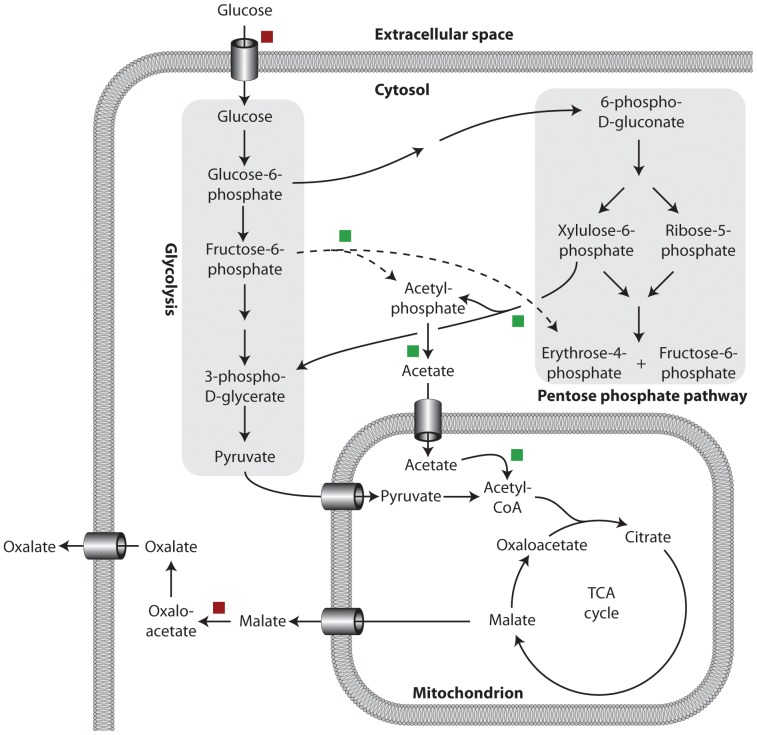
Metabolic map illustrating the response of central carbon metabolism caused by the *oafA* deletion. A red box indicates up-regulation of a gene encoding an enzyme catalyzing this reaction. Green box indicates down-regulation. The dotted line indicates fructose-6-phosphate phosphoketolase, a reaction not previously described within the fungal kingdom.

The product in common for the two phosphoketolase enzymes is acetyl-phosphate that can be converted to acetyl-CoA through two enzymatic steps catalyzed by acetate-kinase and acetyl-CoA synthase, respectively. Together with the two genes encoding the phosphoketolases, the genes encoding acetate-kinase and acetyl-CoA synthase were also down-regulated in the *ΔoafA* mutant. The metabolic response caused by the deletion of *oafA* is presented in Figure 4. As a consequence of the down regulation of the phosphoketolases, the acetate-kinase and the acetyl-CoA synthase, the cytosolic pool of acetyl-phosphate and the mitochondrial acetate pool must be reduced. To compensate for this, a larger fraction of mitochondrial AcCoA must originate from pyruvate, which requires a higher flux through glycolysis. This argument is supported by the reported correlation between the glycolytic flux and the pool size of acetyl-CoA [34]. Sorensen et al. (2009) observed that addition of lactate, led to an increased pool of AcCoA, which caused an up-regulation of the rate-limiting enzyme of the pentose phosphate pathway (PP pathway), Glucose-6-phosphate dehydrogenase [34]. In addition, increased flux through glycolysis has earlier been associated with increased organic acid production. In citrate overflow metabolism, the majority of the citrate produced originates from the glycolytic pathway [35], [36]. However, based on the transcriptional profiling of the *ΔoafA* mutant, none of the genes encoding glycolytic enzymes were up-regulated. As described earlier, an up-regulation of sugar permeases were argued to mediate an increased reuptake of gluconate in the *ΔoafA* mutant compared to the wild type strain. Furthermore, since the cultures were carried out as glucose limited chemostats, the glucose uptake rate was fixed but the reuptake of gluconate by the mutant strain could result in a higher amount of carbon metabolized through the glycolysis without a significant up-regulation of the glycolytic genes, subsequently leading to the observed overproduction of oxalate.

In continuation of the proposed higher flux through glycolysis, the measured down-regulation of the two phosphoketolase genes indicate a lower flux through the pentose phosphate pathway in the *oafA* mutant compared to the wild type strain. The primary outcome of the PP-pathway is generation of reducing equivalents in the form of NADPH, used mainly in reductive biosynthesis reactions *e.g.* amino acid biosynthesis. A reduced flux through the PP-pathway would therefore result in a lower biomass yield and this further substantiates the argument of a lower PP-pathway in the *oafA* mutant, as the biomass yield, Y_SX_; in the *ΔoafA* strain was reduced compared to Y_SX_ of the wild type. Finally, in the reverse study where *oahA* was deleted in *A. niger* eliminating oxalic acid production, an increase in the PP-pathway flux of 10% was detected [28], [37]. Whether this was due to an up-regulation of the phosphoketolases can only be speculative since neither transcription profiles nor enzyme activities of these two enzymes were measured. To summarize, based on the results of the transcriptional profiles and the physiological characterizations of the *oafA* mutant and the wild type strain, it is argued that the increased oxalate formation in the *oafA* mutant is a consequence of an effective re-uptake of gluconate and thereby a higher flux through glycolysis resulting in a lower flux through the PP-pathway, demonstrated by down-regulation of the two phosphoketolases.

### Oxidative phosphorylation

The oxidative phosphorylation is responsible for the major ATP generation during aerobic metabolism by coupling reoxidation of NADH to ATP synthesis. In the *ΔoafA* strain, down-regulation of the oxidative phosphorylation was observed both physiologically by a 35% decrease in oxygen consumption per cmol glucose (*Y_so_*) and transcriptionally as down-regulation of a NADH dehydrogenase and monooxygenase, both with ubiquinone binding. The decreased activity of the oxidative phosphorylation is further supported by the down-regulation of genes encoding a catalase and a chloroperoxidase. These enzymes are highly conserved in organisms exposed to oxygen and aid removal of reactive oxygen species that is an inevitable byproduct of the cytochrome c oxidase complex. To our knowledge a connection between oxidative phosphorylation and oxalate production has not been described, however the mutant response presented here provides indications of such a connection.

## Conclusion

From the results described above it is concluded that OafA is a trans-acting transcription factor since deletion of the responsible gene resulted in 241 genes being differently expressed compared to the wild type strain. In addition, the function of OafA as a transcription factor was further underlined by the altered acid production profile in the *ΔoafA* mutant strain, with focus on the mutant being an oxalate overproducing strain.

Through transcription analysis, reduced phosphoketolase activity together with increased reuptake of gluconate were identified as being the main players in the metabolic response, resulting in the observed oxalate overproducing phenotype of the *ΔoafA* strain. Furthermore, the oxidative phosphorylation was down-regulated in the *oafA* mutant and this indicates an interesting correlation between the oxidative phosphorylation and organic acid formation.

## Supporting Information

Table S1 The raw data for the expression level of all analyzed *A. niger* genes. In addition, complete lists of the significantly regulated genes at the two different pH's are also presented.
(XLSX)

## References

[1] Baker SE (2006) *Aspergillus niger* genomics: past, present and into the future. Med Mycol 44 Suppl 1: S17–21. 17050415 10.1080/13693780600921037

[2] Schuurmans JM, Rossell SL, van Tuijl A, Bakker BM, Hellingwerf KJ, et al (2008) Effect of hxk2 deletion and HAP4 overexpression on fermentative capacity in *Saccharomyces cerevisiae* . FEMS Yeast Res 8: 195–203. 18179578 10.1111/j.1567-1364.2007.00319.x

[3] Alper H, Moxley J, Nevoigt E, Fink GR, Stephanopoulos G (2006) Engineering yeast transcription machinery for improved ethanol tolerance and production. Science 314: 1565–1568. 17158319 10.1126/science.1131969

[4] Goffeau A, Barrell BG, Bussey H, Davis RW, Dujon B, et al (1996) Life with 6000 genes. Science 274: 546, 563–547. 8849441 10.1126/science.274.5287.546

[5] Galagan JE, Calvo SE, Borkovich KA, Selker EU, Read ND, et al (2003) The genome sequence of the filamentous fungus Neurospora crassa. Nature 422: 859–868. 12712197 10.1038/nature01554

[6] McCue LA, Thompson W, Carmack CS, Lawrence CE (2002) Factors influencing the identification of transcription factor binding sites by cross-species comparison. Genome Res 12: 1523–1532. 12368244 10.1101/gr.323602 PMC187528

[7] Pel HJ, de Winde JH, Archer DB, Dyer PS, Hofmann G, et al (2007) Genome sequencing and analysis of the versatile cell factory *Aspergillus niger* CBS 513.88. Nat Biotechnol 25: 221–231. 17259976 10.1038/nbt1282

[8] Milo R, Shen-Orr S, Itzkovitz S, Kashtan N, Chklovskii D, et al (2002) Network motifs: simple building blocks of complex networks. Science 298: 824–827. 12399590 10.1126/science.298.5594.824

[9] Schumann J, Hertweck C (2006) Advances in cloning, functional analysis and heterologous expression of fungal polyketide synthase genes. J Biotechnol 124: 690–703. 16716432 10.1016/j.jbiotec.2006.03.046

[10] Nielsen ML, Nielsen JB, Rank C, Klejnstrup ML, Holm DK, et al (2011) A genome-wide polyketide synthase deletion library uncovers novel genetic links to polyketides and meroterpenoids in *Aspergillus nidulans* . FEMS Microbiol Lett 321: 157–166. 21658102 10.1111/j.1574-6968.2011.02327.x

[11] Punt PJ, Schuren FH, Lehmbeck J, Christensen T, Hjort C, et al (2008) Characterization of the *Aspergillus niger* prtT, a unique regulator of extracellular protease encoding genes. Fungal Genet Biol 45: 1591–1599. 18930158 10.1016/j.fgb.2008.09.007

[12] Ruijter GJ, van de Vondervoort PJ, Visser J (1999) Oxalic acid production by *Aspergillus niger:* an oxalate-non-producing mutant produces citric acid at pH 5 and in the presence of manganese. Microbiology 145 Pt 9: 2569–2576. 10517610 10.1099/00221287-145-9-2569

[13] Heinrich M, Rehm HJ (1982) Formation of gluconic acid at low pH-values by free and immobilized *Aspergillus niger* cells during citric acid fermentation. Applied Microbiology and Biotechnology 15: 88–92.

[14] Witteveen FB, van de Vondervoort PJ, van den Broeck HC, van Engelenburg AC, de Graaff LH, et al (1993) Induction of glucose oxidase, catalase, and lactonase in *Aspergillus niger* . Curr Genet 24: 408–416. 8299156 10.1007/BF00351849

[15] Andersen MR, Lehmann L, Nielsen J (2009) Systemic analysis of the response of *Aspergillus niger* to ambient pH. Genome Biol 10: R47. 19409083 10.1186/gb-2009-10-5-r47 PMC2718513

[16] Nielsen ML, Albertsen L, Lettier G, Nielsen JB, Mortensen UH (2006) Efficient PCR-based gene targeting with a recyclable marker for *Aspergillus nidulans* . Fungal Genet Biol 43: 54–64. 16289954 10.1016/j.fgb.2005.09.005

[17] McCluskey K, Wiest A, Plamann M (2010) The Fungal Genetics Stock Center: a repository for 50 years of fungal genetics research. J Biosci 35: 119–126. 20413916 10.1007/s12038-010-0014-6

[18] Andersen MR, Salazar MP, Schaap PJ, van de Vondervoort PJ, Culley D, et al (2011) Comparative genomics of citric-acid-producing *Aspergillus niger* ATCC 1015 versus enzyme-producing CBS 513.88. Genome Res 21: 885–897. 21543515 10.1101/gr.112169.110 PMC3106321

[19] Sambrook J, Russell DW (2001) Molecular cloning: a laboratory manual. Cold Spring Harbor, N.Y.: Cold Spring Harbor Laboratory Press.

[20] Smyth GK (2004) Linear models and empirical bayes methods for assessing differential expression in microarray experiments. Stat Appl Genet Mol Biol 3: Article3. 16646809 10.2202/1544-6115.1027

[21] Irizarry RA, Bolstad BM, Collin F, Cope LM, Hobbs B, et al (2003) Summaries of Affymetrix GeneChip probe level data. Nucleic Acids Res 31: e15. 12582260 10.1093/nar/gng015 PMC150247

[22] Benjamini Y, Hochberg Y (1995) Controlling the False Discovery Rate: A Practical and Powerful Approach to Multiple Testing. Journal of the Royal Statistical Society Series B (Methodological) 57: 289–300.

[23] McIntyre M, McNeil B (1997) Effect of carbon dioxide on morphology and product synthesis in chemostat cultures of *Aspergillus niger* A60. Enzyme and Microbial Technology 21: 479–483.

[24] Muller C, McIntyre M, Hansen K, Nielsen J (2002) Metabolic engineering of the morphology of *Aspergillus oryzae* by altering chitin synthesis. Appl Environ Microbiol 68: 1827–1836. 11916702 10.1128/AEM.68.4.1827-1836.2002 PMC123896

[25] Reyes-Dominguez Y, Bok JW, Berger H, Shwab EK, Basheer A, et al (2010) Heterochromatic marks are associated with the repression of secondary metabolism clusters in *Aspergillus nidulans* . Mol Microbiol 76: 1376–1386. 20132440 10.1111/j.1365-2958.2010.07051.x PMC2904488

[26] Nutzmann HW, Reyes-Dominguez Y, Scherlach K, Schroeckh V, Horn F, et al (2011) Bacteria-induced natural product formation in the fungus *Aspergillus nidulans* requires Saga/Ada-mediated histone acetylation. Proc Natl Acad Sci U S A 108: 14282–14287. 21825172 10.1073/pnas.1103523108 PMC3161617

[27] Shwab EK, Bok JW, Tribus M, Galehr J, Graessle S, et al (2007) Histone deacetylase activity regulates chemical diversity in *Aspergillus* . Eukaryot Cell 6: 1656–1664. 17616629 10.1128/EC.00186-07 PMC2043372

[28] Pedersen H, Hjort C, Nielsen J (2000) Cloning and characterization of oah, the gene encoding oxaloacetate hydrolase in *Aspergillus niger* . Mol Gen Genet 263: 281–286. 10778746 10.1007/s004380051169

[29] Ma H, Kubicek CP, Röhr M (1981) Malate dehydrogenase isoenzymes in *Aspergillus niger* . FEMS Microbiology Letters 12: 147–151.

[30] de Jongh WA, Nielsen J (2008) Enhanced citrate production through gene insertion in *Aspergillus niger* . Metab Eng 10: 87–96. 18162426 10.1016/j.ymben.2007.11.002

[31] Thykaer J, Nielsen J (2007) Evidence, through C13-labelling analysis, of phosphoketolase activity in fungi. Process Biochemistry 42: 1050–1055.

[32] Roopashri AN, Varadaraj MC (2009) Molecular characterization of native isolates of lactic acid bacteria, bifidobacteria and yeasts for beneficial attributes. Appl Microbiol Biotechnol 83: 1115–1126. 19407995 10.1007/s00253-009-1991-y

[33] Ingram-Smith C, Martin SR, Smith KS (2006) Acetate kinase: not just a bacterial enzyme. Trends Microbiol 14: 249–253. 16678422 10.1016/j.tim.2006.04.001

[34] Sorensen LM, Lametsch R, Andersen MR, Nielsen PV, Frisvad JC (2009) Proteome analysis of *Aspergillus niger*: lactate added in starch-containing medium can increase production of the mycotoxin fumonisin B2 by modifying acetyl-CoA metabolism. BMC Microbiol 9: 255. 20003296 10.1186/1471-2180-9-255 PMC2807875

[35] Cleland WW, Johnson MJ (1954) Tracer experiments on the mechanism of citric acid formation by *Aspergillus niger* . J Biol Chem 208: 679–689. 13174578

[36] Legiša M, Mattey M (1986) Glycerol as an initiator of citric acid accumulation in *Aspergillus niger* . Enzyme and Microbial Technology 8: 258–259.

[37] Pedersen H, Christensen B, Hjort C, Nielsen J (2000) Construction and Characterization of an Oxalic Acid Nonproducing Strain of *Aspergillus niger* . Metabolic Engineering 2: 34–41. 10935933 10.1006/mben.1999.0136

